# Validity and reliability of a wearable blood flow restriction training device for arterial occlusion pressure assessment

**DOI:** 10.3389/fphys.2024.1404247

**Published:** 2024-06-07

**Authors:** Wei-Yang Zhang, Shu-Can Zhuang, Yuan-Ming Chen, Hao-Nan Wang

**Affiliations:** ^1^ School of Sports Medicine and Health, Chengdu Sport University, Chengdu, Sichuan, China; ^2^ Sports Medicine Key Laboratory of Sichuan Province, Chengdu, Sichuan, China; ^3^ Sports Medicine Center, West China Hospital, Sichuan University, Chengdu, Sichuan, China; ^4^ Department of Orthopedics and Orthopedic Research Institute, West China Hospital, Sichuan University, Chengdu, Sichuan, China

**Keywords:** blood flow restriction, arterial occlusion pressure, strength training, muscle training, Doppler ultrasound

## Abstract

**Purpose:**

The blood flow restriction (BFR) training is an effective approach to promoting muscle strength, muscle hypertrophy, and regulating the peripheral vascular system. It is recommended to use to the percentage of individual arterial occlusion pressure (AOP) to ensure safety and effectiveness. The gold standard method for assessing arterial occlusive disease is typically measured using Doppler ultrasound. However, its high cost and limited accessibility restrict its use in clinical and practical applications. A novel wearable BFR training device (Airbands) with automatic AOP assessment provides an alternative solution. This study aims to examine the reliability and validity of the wearable BFR training device.

**Methods:**

Ninety-two participants (46 female and 46 male) were recruited for this study. Participants were positioned in the supine position with the wearable BFR training device placed on the proximal portion of the right thigh. AOP was measured automatically by the software program and manually by gradually increasing the pressure until the pulse was no longer detected by color Doppler ultrasound, respectively. Validity, inter-rater reliability, and test-retest reliability were assessed by intraclass correlation coefficients (ICC) and Bland-Altman analysis.

**Results:**

The wearable BFR training device demonstrated good validity (ICC = 0.85, mean difference = 4.1 ± 13.8 mmHg [95% CI: −23.0 to 31.2]), excellent inter-rater reliability (ICC = 0.97, mean difference = −1.4 ± 6.7 mmHg [95% CI: −14.4 to 11.7]), and excellent test-retest reliability (ICC = 0.94, mean difference = 0.6 ± 8.6 mmHg [95% CI: −16.3 to 17.5]) for the assessment of AOP. These results were robust in both male and female subgroups.

**Conclusion:**

The wearable BFR training device can be used as a valid and reliable tool to assess the AOP of the lower limb in the supine position during BFR training.

## 1 Introduction

Resistance training is a fundamental component of exercise programs due to its effectiveness in increasing muscle strength, promoting muscle hypertrophy (Hughes et al., 2017). In addition, resistance training has beneficial effects on cardiovascular function and angiogenesis (Gavin et al., 2007; Gomes et al., 2018). According to the recommendations of the American College of Sports Medicine, promoting muscle hypertrophy and increasing muscle strength requires a minimum resistance load of 70% and 60% of an individual’s one-repetition maximum (1RM) (American College of Sports Medicine position stand, 2009). However, high-load resistance training may not be tolerated in patients with pre-existing joint deterioration or elderly population due to the high mechanical stress and cardiovascular risk involved (Jan et al., 2008; Vale et al., 2018).

Blood flow restriction (BFR) combined with low-load resistance training (LLRT) at intensities of 20%–30% of 1RM is gaining increasing interest in strength training research and clinical practice. The BFR technique involves using a pneumatic cuff or tourniquet around the proximal limb to partially obstruct arterial blood flow (Iida et al., 2011). This technique induces local ischemia and hypoxia, enhancing physiological metabolic stress (Takarada et al., 1985a; Reeves et al., 1985) and activating type II muscle fibers during exercise (Moore et al., 2004). Furthermore, the BFR reduces blood flow and oxygen delivery, resulting in increased angiogenesis-related gene expression (e.g., HIF-1α and VEGF) in skeletal muscle (Larkin et al., 2012). The upregulated gene expression of angiogenesis is closely related to post-exercise angiogenesis and increased capillary growth (Hunt et al., 1985). Due to these effects, BFR combined with LLRT produces muscle size and strength adaptations that are similar to those of high-load resistance training (HLRT) (Centner et al., 2019). Consequently, BFR combined with LLRT could be a feasible and substitute method to HLRT, especially for patients with injuries or elderly population (Wang et al., 2022a; Li et al., 2023).

The BRF technique involves several parameters that significantly affect training adaptations and require careful consideration. These parameters include resistance load, volume, cuff pressure, cuff width, duration of BFR, and the form of BFR (continuous or intermittent). Among these parameters, cuff pressure is considered a critical determinant for achieving optimal training adaptations and ensuring safety. Studies have shown that acute physiological responses and chronic physiological adaptations are pressure-dependent. Higher cuff pressures lead to increased metabolic stress and muscle stimuli (Loenneke et al., 2014; Counts et al., 2016). In the past, studies have used arbitrary absolute cuff pressures in BFR training, such as 100 mmHg (Takarada et al., 1985b; Takarada et al., 2000). However, this approach has been criticized because it does not consider the characteristics of the cuff, blood pressure, or limb circumference (Loenneke et al., 2012; Loenneke et al., 2013a; Loenneke et al., 2015; Jessee et al., 2016). Applying the same absolute pressure to different individuals may lead to inconsistent BFR levels. Ensuring that all participants in a group receive a similar BFR stimulus is essential for consistency in research (Jessee et al., 2016; Wang et al., 2022b). In clinical practice, it is crucial to avoid excessively high pressures due to potential safety concerns (Patterson et al., 2019).

Therefore, it is recommended to use the relative cuff pressure to produce a similar BFR stimulus in the personalized BFR prescription. Relative cuff pressure is determined as a preconfigured percentage of the Arterial Occlusion Pressure (AOP), which is the minimum pressure required to completely block arterial blood flow to the distal limb (Patterson et al., 2019). Measurement of AOP is necessary because the relative pressure of AOP affects local limb ischemia and microvascular oxygenation (Reis et al., 2019). Typically, AOP is measured by a Doppler ultrasound device in conjunction with an automatic or manual gradual inflation tourniquet system (Loenneke et al., 2013b). However, specialized instruments for BFR training are expensive and primarily located in research laboratories. Additionally, performing AOP assessments by Doppler ultrasound device require specialized training and experience (Karanasios et al., 2022), which can be a limitation when performed by professionals without prior experience. Although there is substantial research supporting the benefits of BFR training in muscle and peripheral vascular system (Giles et al., 2017; Hughes et al., 2017), implementing it in outdoor, clinical and home settings remains challenging.

Recently, BFR training devices have incorporated partial circumferential bladder designs, which has led to the development of wearable BFR training devices (Rolnick et al., 2023). The ability to detect AOP in wearable BFR training device simplifies the use of BFR training in outdoor, rehabilitation clinics and home settings. However, further studies are required to determine the validity and reliability of these wearable BFR training devices in detecting AOP, as they are crucial for the device’s effectiveness and safety in BFR training. The aim of this study was to evaluate the validity, inter-rater reliability, and test-retest reliability of the wearable BFR training device. We hypothesized that the wearable BFR training device demonstrates good validity, inter-rater reliability and test-retest reliability in detecting AOP of lower limb.

## 2 Materials and methods

### 2.1 Participants

A power analysis considering repeated measures in each subject was performed prior to the study. A sample size of 92 subjects was determined, with a significance level of 0.05, power of 0.80, P0 (ICC) = 0.8, P1 (ICC) = 0.9, and subgroup = 2 (Walter et al., 1998). Ninety-two participants (46 females and 46 males) were recruited for the current study. The study participants were healthy individuals between the ages of 18 and 45 with no history of coagulation disorders, including deep vein thrombosis. They engaged in at least 30 min of moderate-intensity physical activity three times per week, as defined by the Godin Leisure-Time Physical Activity Questionnaire (Godin and Shephard, 1997). Exclusion criteria for this study included pregnancy, smoking, lymphoedema, cardiovascular disease, chronic degenerative diseases, a history of cancer, recent surgery in the lower limbs within the past 12 months, or medication use affecting blood flow regulation.

### 2.2 Study design and procedures

All participants were required to sign a written informed consent form prior to participation, which described the purpose and risks of the study in accordance with the standards of the Declaration of Helsinki. The study protocol was approved by the Ethics Committee of Chengdu Sports University (Approval No. 2022-78).

**FIGURE 1:**
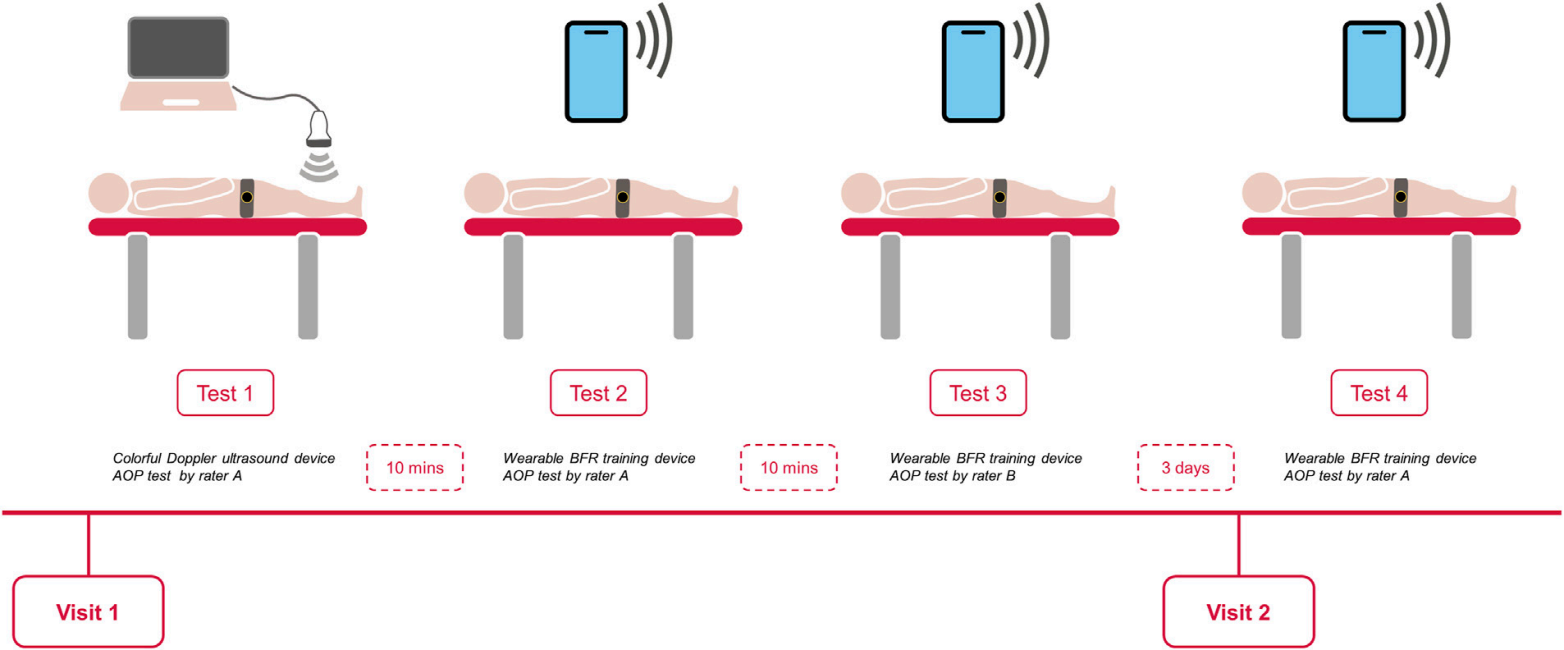
Schematic diagram of the process of the arterial occlusion pressure test.

All eligible participants visited the same laboratory twice during the experiment. During the first visit, an independent assessor recorded their demographic characteristics and anthropometric data, including age, body mass, height, brachial blood pressure, and the mid-thigh circumference. The AOP was then assessed using the color Doppler ultrasound device and the wearable BFR training device, respectively. The order of the tests on the first day was randomized, and the interval time between each test was 10 min. The During the second visit, which occurred 3 days after the first visit (Karanasios et al., 2022), the AOP was measured solely by the wearable BFR training device (Figure 1). The measurements were performed in a quiet, temperature-controlled room (22°C [1°C]). Participants were instructed to abstain from high-intensity activity, caffeine, and alcohol for at least 24 h prior to the visits.

The mid-thigh circumference was measured following the guidelines of the International Society for the Advancement of Kinanthropometry (Esparza-Ros et al., 2019). The midpoint of the thigh was marked between the inguinal crease and the midpoint of the posterior superior border of the patella while the participant was in an upright sitting position. The circumference of the dominant leg was then measured at the midpoint of the thigh using a steel measuring tape while the participant was in a relaxed standing position. Participants were instructed to rest in the supine position for 10 min prior to blood pressure measurement. Systolic blood pressure (SBP) and diastolic blood pressure (DBP) were measured at the brachial artery using an electronic device (HEM-7132, OMRON, Dalian, China) and recorded (Lacruz et al., 2017).

### 2.3 Determination of AOP by wearable BFR training device

The AOP measurements were obtained using the woven fabric and plastic cuff (56 × 10 cm; Airbands, VALD Health, Brisbane, Australia) with an electronic pump system. The cuff was placed on the most proximal portion of the thigh (25% of the femur length) and secured with a snug fit (Sieljacks et al., 2018). To ensure hemodynamic normalization, a rest period of 10 min was provided before determining AOP. The measurement was performed using the software program of the device in the “Automatic Mode.” In this mode, the device automatically inflates gradually to detect the AOP and deflates after testing.

### 2.4 Determination of AOP by color Doppler ultrasound

To determine AOP using color Doppler ultrasound, the cuff was placed in the same position on the thigh as previously described (Wang et al., 2022b). The portable color Doppler ultrasound (CX50, Philips Healthcare, Best, Netherlands) was positioned at the ankle to measure the pedal pulse. The measurement was performed using the software program in the “Manual Pressure Mode.” The cuff pressure was initially inflated to 50 mmHg and then gradually increased in 10 mmHg increments until the pedal pulse disappeared on the color Doppler ultrasound (Zeng et al., 2019). At this point, the pressure was assumed to represent a total arterial occlusion and recorded. The pressure was then manually deflated. All measurements were made using the same equipment.

### 2.5 Validity

The raters were well-trained physiotherapists in the field of musculoskeletal disorders, and had the experience with the BFR training technique. To assess the validity, rater A measured the AOP of each participant using the color Doppler ultrasound device (Test 1) and wearable BFR training device (Test 2) during the first visit. The results of Test 1 and Test 2 were used to evaluate the validity of the wearable BFR training device. Rater A had the experience with the color Doppler ultrasound device and operated it independently. The disappearance of the pedal pulse was determined by the rater A, and another rater recorded the AOP. The test procedure required the rater to complete each test with three measurements, with an interval time of 5 min between each measurement, and the average value of these three measurements was used for statistical analysis.

### 2.6 Reliability

For the inter-rater reliability, two raters measured the AOP of each participant using a wearable BFR training device during the first visit. The AOP assessed by the Rater B (Test 3) each participant was compared with AOP assessed by the Rater A (Test 2). To avoid the assessor bias, the raters were blinded to the AOP that was recorded from the other rater. For the test-retest reliability, rater A (Test 4) reassessed each participant on the second visit, which was 3 days after the first measurement. The AOP was also recorded by the other raters. Also, the test procedure required the raters to complete each test three times, and the average value of the three attempts will be used in the statistical analysis. The rater order remained the same for all subjects.

### 2.7 Statistical analysis

All statistical analyses were performed using IBM SPSS Statistics 26.0 (SPSS Inc., Armonk, NY, United States). Demographic characteristics and anthropometric data were presented as mean and standard deviation (SD). The normality of distribution was assessed using the Shapiro-Wilk test, and all continuous data were found to follow a normal distribution. The validity (Test 1 and Test 2) of the AOP measurement was determined by calculating ICC with 95% CI in a two-way random effect model. The inter-rater reliability (Test 2 and Test 3) and test-retest reliability (Test 2 and Test 4) of the AOP measurement were determined by calculating intraclass correlation coefficients (ICC) with 95% confidence intervals (CI) in a two-way mixed effect model. ICC values below 0.5 indicate poor reliability, values between 0.5 and 0.75 indicate moderate reliability, values between 0.75 and 0.9 indicate good reliability, and values above 0.90 indicate excellent reliability (Hopkins et al., 2009). The Bland-Altman plots were used to indicate the systematic error and level of agreement in validity, inter-reliability, and test-retest reliability. In addition, we divided all of the participants into two subgroups according to gender, which were the male subgroup and the female subgroup. Pearson’s product moment correlation coefficient analysis was used to assess the relationship between AOP and potential predictive factors. Cuff pressures measured by color Doppler ultrasound were used for these calculations. Correlations are categorized as having a large effect with coefficients greater than 0.5, a moderate effect with coefficients around 0.3, and a small association with values greater than 0.1 (Cohen, 1992). The significance level was set at 0.05.

## 3 Results

All 92 participants completed the study, and no adverse events or dropouts were reported. Descriptive and anthropometric characteristics data of the participants at the baseline were shown in Table 1.

**TABLE 1:** Demographics and characteristics of the participants.

	Total (N = 92)			Male (N = 46)			Female (N = 46)		
	Mean ± SD	Min	Max	Mean ± SD	Min	Max	Mean ± SD	Min	Max
Age	23.3 ± 2.9	18.0	30.0	23.4 ± 2.9	18.0	29.0	23.2 ± 2.8	18.0	30.0
Height (cm)	171.3 ± 8.6	153.1	193.1	177.7 ± 5.4	168.7	193.1	164.9 ± 5.9	153.1	179.4
Body mass (kg)	63.0 ± 8.8	47.9	85.8	69.9 ± 5.8	60.1	85.8	56.2 ± 5.3	47.9	68.1
BMI (kg/m^2^)	21.4 ± 1.6	17.2	23.9	22.1 ± 1.2	19.5	23.9	20.7 ± 1.5	17.2	23.8
Systolic blood pressure (mmHg)	117.5 ± 11.7	91.0	138.0	123.9 ± 7.6	110.0	138.0	111.0 ± 11.6	91.0	136.0
Diastolic blood pressure (mmHg)	71.9 ± 7.0	60.0	90.0	72.5 ± 7.1	60.0	90.0	71.3 ± 6.9	60.0	88.0
Thigh circumference (cm)	51.5 ± 4.4	41.6	62.4	52.9 ± 4.2	44.6	62.4	50.0 ± 4.1	41.6	60.2

### 3.1 Validity

**FIGURE 2:**
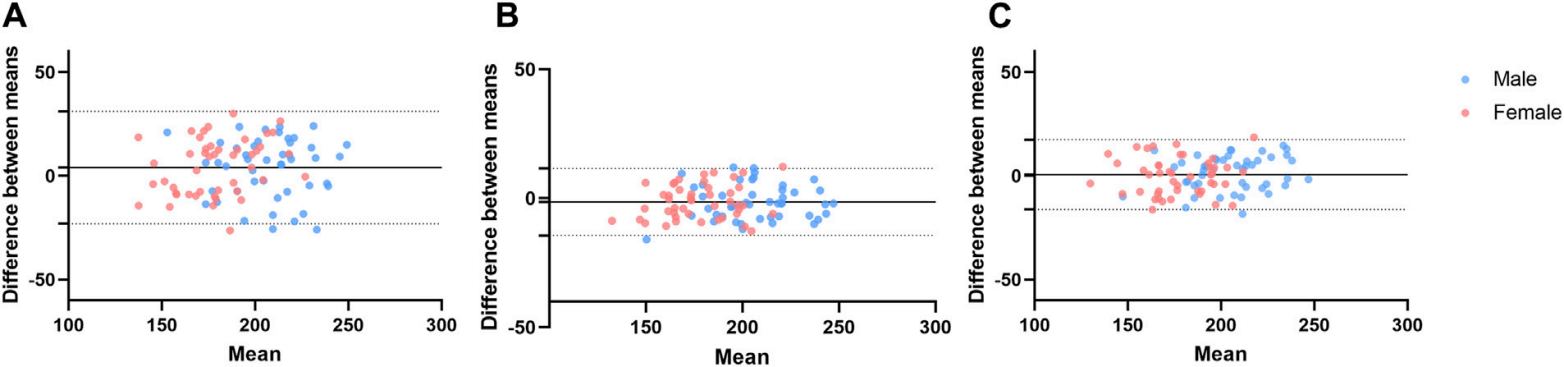
Bland-Altman plots of the arterial occlusion pressure for validity **(A)**, inter-rater reliability **(B)**, and test-retest reliability **(C)**. The dots represent individual participants for male (blue) and female (pink). The dotted line indicates the ±1.96 standard deviations of the differences from the mean difference.

The AOP of the lower limb measured by the color Doppler ultrasound and the wearable BFR training device was 195.4 ± 26.9 mmHg and 191.4 ± 25.8 mmHg. According to the Bland-Altman plot (Figure 2), the mean difference between the two methods was 4.1 ± 13.8 mmHg (95% CI of Limits of Agreement: −23.0–31.2) between the two methods. The ICC value of the validity was 0.85 (Table 2). Therefore, the wearable BFR training device showed good validity for measuring AOP at the lower limb.

**TABLE 2:** Validity, inter-rater reliability, and test-retest reliability of wearable BFR training device.

		Total (N = 92)	Male (N = 46)	Female (N = 46)
Validity	ICC (95% CI)	0.85 (0.78, 0.90)	0.79 (0.64, 0.88)	0.80 (0.66, 0.89)
	Mean difference ±SD (mm Hg)	4.1 ± 13.8	3.7 ± 14.3	4.5 ± 13.5
Inter-rater reliability	ICC (95% CI)	0.97 (0.95, 0.98)	0.95 (0.92, 0.97)	0.94 (0.90, 0.97)
	Mean difference ±SD (mm Hg)	−1.4 ± 6.7	−1.5 ± 6.8	−1.3 ± 6.6
Test-retest reliability	ICC (95% CI)	0.94 (0.91, 0.96)	0.93 (0.87, 0.96)	0.90 (0.83, 0.95)
	Mean difference ±SD (mm Hg)	0.6 ± 8.6	1.6 ± 8.4	−0.3 ± 8.9

In the subgroup analysis, the Bland-Altman plot (Figure 2), the mean difference between the color Doppler ultrasound and the wearable BFR training device was 3.7 ± 14.3 mmHg (95% CI of Limits of Agreement: −24.3–31.7) for males and 4.5 ± 13.5 mmHg (95% CI of Limits of Agreement: −21.9–30.8) for females, respectively. The validity of measuring AOP at the lower limb between the color Doppler Ultrasound and the wearable BFR training device was excellent for both male (ICC = 0.79 and female (ICC = 0.80).

### 3.2 Inter-rater reliability

The AOP of the lower limb, as measured by another rater, was 192.7 ± 25.4 mmHg. Bland-Altman plot (Figure 2) showed that the mean difference between the two raters was −1.4 ± 6.7 mmHg (95% CI of Limits of Agreement: −14.4 to 11.7). The ICC value of the inter-rater reliability was 0.97 (Table 2). Therefore, the wearable BFR training device showed excellent inter-rater reliability for measuring AOP at the lower limb.

The subgroup analysis revealed the inter-rater reliability for gender, and the Bland-Altman plot (Figure 2) indicated that the mean difference between two raters was −1.5 ± 6.8 mmHg (95% CI of Limits of Agreement: −14.8 to 11.9) for males and −1.3 ± 6.6 mmHg (95% CI of Limits of Agreement: −14.1 to 11.6) for females, respectively. The inter-rater reliability of measuring AOP at the lower limb was excellent for both male (ICC = 0.95) and female (ICC = 0.94) subgroups.

### 3.3 Test-retest reliability

The results indicated that the AOP tested by the wearable BFR training device was 190.8 ± 24.6 mmHg for the second visit, respectively. According to the Bland-Altman plot (Figure 2), the mean difference of AOP between the two visits was 0.6 ± 8.6 mmHg (95% CI of Limits of Agreement: −16.3–17.5). The ICC value of the test-retest reliability was 0.94 (Table 2). Therefore, the wearable BFR training device demonstrated excellent test-retest reliability for measuring AOP at the lower limb.

In the subgroup analysis, we discriminated test-retest reliability for gender. According to the Bland-Altman plot (Figure 2), the mean difference of AOP between two visits was 1.6 ± 8.4 mmHg (95% CI of Limits of Agreement: −14.9–17.9) for males and −0.3 ± 8.9 mmHg (95% CI of Limits of Agreement: −17.7 to 17.0) for females, respectively. The test-retest reliability of measuring AOP at the lower limb was excellent for both male (ICC = 0.93) and female (ICC = 0.90) subgroups.

### 3.4 Correlation analysis

**FIGURE 3:**
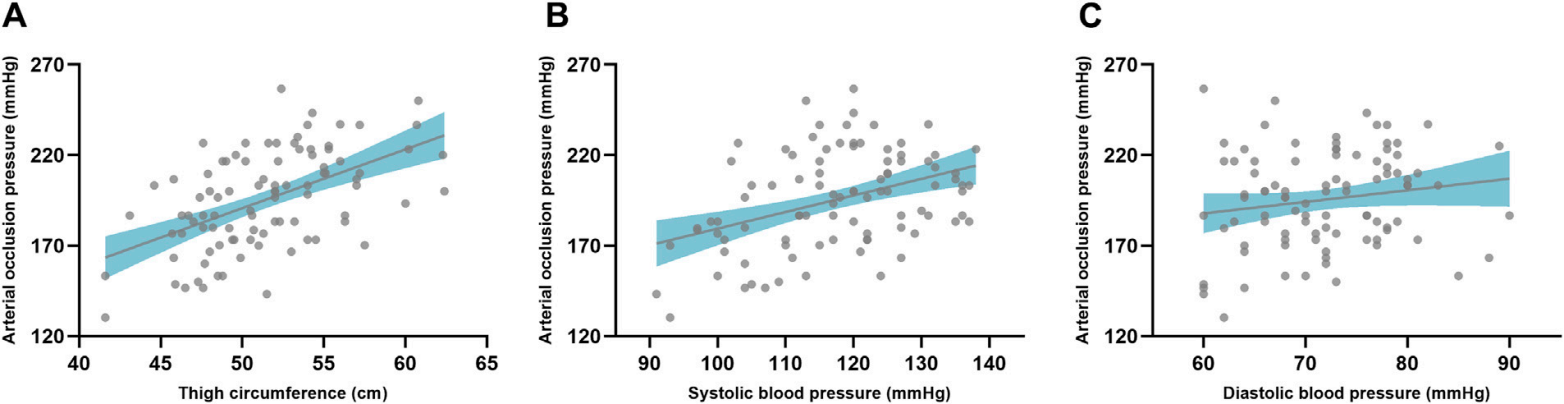
The correlation between limb circumference and arterial occlusion pressure **(A)**. The correlation between arterial occlusion pressure and blood pressure with SBP **(B)** and DBP **(C)**, respectively. The gray dot represents the individual data and the gray line indicates the regression line with the confidence interval (shaded area).

The correlation analyses indicated there was significant correlation (*p* < 0.01) with a large effect (r = 0.53) between thigh circumference and AOP when using the color Doppler ultrasound device (Figure 3). Additionally, SBP was significantly correlated (*p* < 0.01) with AOP when using the color Doppler ultrasound device with moderate effect (r = 0.40). However, there was no significant correlation found between the DBP and AOP when using the color Doppler ultrasound device (*p* = 0.17).

## 4 Discussion

The results of this research contribute to our understanding of measuring AOP by using a wearable BFR training device. We reported good validity in the measures of AOP using the color Doppler ultrasound device and wearable BFR training device in the lower limb. We also reported that the wearable BFR training device has excellent inter-rater reliability and test-retest reliability for measuring AOP in the lower limb. Furthermore, the subgroup analyses indicated that the validity, inter-rater reliability, and test-retest reliability were robust in both males and females. Noteworthy findings are that thigh circumference and SBP, but not DBP, were significantly correlated with AOP in the lower limb.

The wearable BFR training device comprised of a partial circumferential bladder tourniquet and a personalized tourniquet instrument with AOP calculation sensors (Rolnick et al., 2023). The cuff was placed on the patient’s limb, and the pneumatic system connected to the tourniquet cuff, increasing the cuff pressure in incremental steps. During this process, the pressure sensor analyzed the pneumatic pressure pulsations in the cuff bladder by the arterial pressure pulsations at each cuff pressure increment, and used these characteristics to determine AOP (McEwen et al., 2015). The results of our study showed good validity between the color Doppler Ultrasound and the wearable BFR training device (mean difference 4.1 mmHg). Numerous attempts have been made to find more convenient methods for measuring AOP, which would expand the practical and clinical applications of BFR techniques. McEwen et al. proposed an automated method for measuring AOP based on a distal transducer that uses a photoplethysmography sensor placed on the most distal phalanx of the affected limb to measure AOP. Zeng et al. (Zeng et al., 2019) evaluated the validity between Doppler ultrasound and pulse oximetry measurements for AOP, and found the difference was not acceptable (mean difference 10.59 mmHg) between these two methods in the lower limb. In addition, automatic pneumatic tourniquet device has developed to measure the individual AOP automatically through two different methods. The first method, the “embedded AOP method,” measures AOP by using a dual-purpose (pressure sensor and pneumatic effector) tourniquet cuff to monitor arterial pulsations in an underlying limb by sensing pneumatic pressure pulsations in the cuff while the cuff pressure is gradually increased. Masri et al. (Masri et al., 2016) reported that the mean difference between the embedded AOP method and the manual Doppler ultrasound method was 0 ± 15 mmHg for measuring AOP. The second method, the “distal AOP method,” measures AOP using a photoplethysmography sensor placed on the patient’s distal finger or toe of the operative limb to monitor arterial pulsations as cuff pressure is gradually “increased.” McEwen et al. (McEwen et al., 2015) found that the mean difference between the distal AOP method and the manual Doppler ultrasound method was 0.08 ± 15.03 mmHg for measuring AOP. Hughes et al. (Hughes and McEwen, 2021) demonstrated that clinically acceptable agreement between the embedded and distal methods of AOP measurement. The wearable BFR training device used in this study applies the embedded AOP method, but is simpler and more portable. Although the accuracy of measuring AOP with the wearable BFR training device was lower than with the automatic pneumatic tourniquet device reported by Masri et al. (Masri et al., 2016), this difference is acceptable. It is important to acknowledge the potential the errors that exist in hemodynamic measurements based on electronic pressure sensors, particularly since device manufacturers often use unpublished algorithms (Bayoumy et al., 2021). For instance, research has been reported that when measuring blood pressure through the oscillometric method, 58% of aneroid sphygmomanometers have been shown to have errors greater than 4 mmHg, with approximately one-third of these having errors greater than 7 mmHg (Mion and Pierin, 1998). Therefore, we suggest that the accuracy of this automated AOP measurement can be further improved by improving the algorithm, especially for wearable devices. To minimize accuracy problems associated with the use of these devices, it is recommended to develop internationally accepted protocols for the validation of automated BFR training devices. These protocols should be referenced to the standards for sphygmomanometersm (O Brien et al., 2001; O Brien et al., 2002). Establishing and adhering to accepted protocols reduces systematic errors and lengthens the time interval between calibrations due to the stability of electrical sensors.

To the best of our knowledge, no study has evaluated the reliability of wearable BFR training devices that use a partial circumferential bladder tourniquet cuff system. Test-retest reliability, which is critical for clinical applications as it helps determine whether we can train with measured arterial occlusion pressures for a given period of time. A previous study demonstrated that handheld Doppler ultrasound with manual pump occlusion cuffs exhibit excellent reliability (ICC = 0.90) (Karanasios et al., 2022). This study found that the wearable BFR training device also had excellent test-retest reliability over a 3-day period. However, the study did not assess test-retest reliability over extended periods to determine calibration intervals for AOP. The test-retest reliability for a longer period of time is unknown. The individualized AOP value may be altered as a result of the adaptation of BFR training. To ensure accuracy, it is recommended to calibrate individual AOP at least every 8 weeks (Mattocks et al., 2019). In addition, the main application scenarios for wearable BFR training device are in non-laboratory settings, such as clinics, gyms, and even at home; and these devices are primarily operated by therapists, coaches, or the users themselves (Cognetti et al., 2022). Consequently, we conducted an inter-tester reliability assessment to determine if measurements by different evaluators yield consistent results. Our findings suggested that the wearable BFR training device had excellent inter-rater reliability with standardized measurement procedures. Trained or instructed assessors can effectively conduct the AOP test using the wearable BFR training device. This also provided the opportunity for patients to complete AOP testing and BFR training using a wearable BFR training device in home scenarios.

To make similar degree of BFR to each participant, individual differences should be considered. Studies that compared AOP between the sexes showed heterogeneous results. For AOP of upper limb, the current findings are relatively consistent that male have been found to have higher AOP than female, whether measured using small (5–6 cm) or medium cuffs (10–13 cm) (Jessee et al., 2016; Brown et al., 2018). For the AOP of the lower limb, gender differences in lower limb AOP were observed only in the use of large cuffs (18 cm), not medium cuffs (13 cm). However, there are also one studies that reported that male had a greater AOP than female when measured using a medium cuff (10 cm). In contrast, there are also studies that report male had a greater AOP than female when measured using a medium cuff (10 cm). One study (Karanasios et al., 2022) identified AOP of lower limb was higher in males only in sitting and standing positions, but not in supine position (Tafunai et al., 2021). One possible explanation of differences between genders is local vascular function. Nishiyama et al. found that female have similar vascular function to men in the upper limbs, but appear to have impaired vascular function in the lower limbs when normalized for shear rate (Nishiyama et al., 2008). It was still controversial whether there is a difference in AOP of the lower limbs, but the influence of gender was considered in this study. Accordingly, the male and female subgroups were further analyzed, and the results were robust for both subgroups with respect to the reliability and validity for measuring AOP by the wearable BFR training device.

The limb circumference influences an individualized AOP. Our results indicated that the thigh circumference was associated with the lower limb AOP with large effect. In the previous study, it was found that the thigh circumference is the strongest predictor of lower limb AOP (Loenneke et al., 2012; Loenneke et al., 2015; Sieljacks et al., 2018). Importantly, the influence of the thigh circumference was not affected by measuring position or cuff width. Hargens et al. (Hargens et al., 1987) found that subcutaneous tissue experiences a greater percentage of the applied pressure compared to deep tissue. The greater the circumference of the limb, the more pronounced this difference in tissue pressure becomes. Therefore, for the same cuff width, a higher inflation pressure would be required to achieve the same deep tissue pressure in a larger limb compared to a smaller limb. After limb circumference, SBP was the next largest predictor of AOP, which was in consistent with a previous study in the lower limb (Loenneke et al., 2015). Similarly, our results indicated a moderate correlation between SBP and AOP in wearable BFR training devices. In contrast, another study by Loenneke et al. (Loenneke et al., 2012) found that DBP, but not SBP, could serve as a predictor of lower limb AOP in the supine position. The wearable BFR training device used in this study utilized a partial circumferential bladder tourniquet, which differs from the full circumferential bladder tourniquet used in previous studies. This difference in tourniquet of BFR training devices may explain the factors influencing occlusion pressure. Although a previous study found an association between the thigh circumference and lower limb AOP in this wearable BFR training device (Keller et al., 2023), it did not investigate the correlation with SBP or DBP. The device had a limited pressure capacity of 270 mmHg. Therefore, its limitations became apparent when enrolling subjects with large thigh circumferences, as larger thigh circumferences have greater AOP. It is important to note that both caffeine and exercise can affect the results of the AOP test by causing hemodynamic changes. Therefore, subjects in this study were asked to rest before the test and to avoid caffeine the day before the experiment to minimize these potential errors. Further studies are needed to investigate the predictors of these novel wearable BFR training devices.

### 4.1 Strength and limitations

In this study, we evaluated the validity, inter-rater reliability, and test-retest reliability of a novel, wearable BFR training device with a partial bladder cuff system. We also investigated the validity, inter-rater reliability, and test-retest reliability of this BFR training device in male and female subgroup. In addition, we analyzed the influencing factors of lower limb AOP within this device, such as thigh circumference and blood pressure. There were several limitations to present study. Firstly, it should be noted that the participants in our study were a young and active population, resulting in relatively small thigh circumference. Previous studies have indicated that the thigh circumference plays a crucial role in the lower limb AOP (Loenneke et al., 2012; Loenneke et al., 2015; Sieljacks et al., 2018). Therefore, our results may not be applicable to all individuals, particularly those with larger thigh circumference and skinfold thickness. Secondly, we did not evaluate the influence of body position on the lower limb AOP while using the wearable BFR training device. Because the influence of body position was not the main purpose of this study. Nevertheless, it was found that the absolute AOP of lower limb in the sitting position is higher than the lower limb AOP in the supine position (Hughes et al., 2018). A previous study reported that the wearable BFR training device may not be able to measure lower limb AOP in some participants due to its limited pressure capacity of 270 mmHg (Keller et al., 2023). Therefore, the sitting position may not be recommended for measuring the AOP using this wearable BFR training device. Finally, the reliability of upper limb AOP in the wearable BFR training device was not assessed in this study due to the difference width between the upper limb cuff and the lower limb cuff. Future studies may evaluate the reliability of upper limb AOP in the wearable BFR training device.

### 4.2 Practical applications

It is necessary to determine the individual AOP before engaging in BFR training. The result of this study indicated that the wearable BFR training device has good validity, excellent inter-rater reliability, as well as excellent test-retest reliability compared to the color Doppler ultrasound. The wearable BFR training device can be used to determine the AOP and relative cuff pressure during BFR training, making it more accessible for both research and practice. The wearable BFR training device provides a solution for the physiotherapist or patient without requiring training in ultrasound techniques. For example, patients can perform BFR training at home with individual cuff pressure under their physiotherapist’s prescription and recommendations for early post-operative rehabilitation. The fact that partial circumferential bladder tourniquet in a wearable BFR training device result in limited applicability for subjects with larger limb circumferences. Currently, the upper limit of cuff pressure for AOP testing by this wearable BFR training device is 270 mmHg, which may not be applicable for subjects with higher AOP subject. These limitations should also be considered when using the wearable BFR training device for both acute and chronic BFR training.

## 5 Conclusion

The wearable BFR training device demonstrated good validity, excellent inter-rater reliability, and excellent test-retest reliability in detecting the AOP at the lower limb. The validity and reliability of wearable BFR training device were consistent across both the male and female groups. Therefore, the wearable BFR training device can be considered a valid and reliable tool for assessing the AOP of the lower limb in the supine position during the BFR training.

## Data Availability

The raw data supporting the conclusion of this article will be made available by the authors, without undue reservation.
